# Achieving sustained virologic response in hepatitis C: a systematic review of the clinical, economic and quality of life benefits

**DOI:** 10.1186/s12879-015-0748-8

**Published:** 2015-01-17

**Authors:** Jayne Smith-Palmer, Karin Cerri, William Valentine

**Affiliations:** Ossian Health Economics and Communications, Ossian Health Economics and Communications GmbH, Bäumleingasse 20, 4051 Basel, Switzerland; Janssen Pharmaceutica NV, Beerse, Belgium

**Keywords:** Sustained virologic response, Chronic hepatitis C, Cost, Quality of life, Utility, Morbidity, Mortality

## Abstract

**Background:**

The goal of chronic hepatitis C treatment is to remove the virus to avoid progression of HCV-related disease. Sustained virologic response (SVR) is the most widely used efficacy endpoint in clinical studies of hepatitis C, and represents the eradication of HCV from the body. The aim of the current review was to examine the long-term clinical, economic and quality of life benefits associated with achieving SVR.

**Methods:**

A systematic literature review was performed using the PubMed, EMBASE and Cochrane library databases to identify articles examining the clinical, economic and quality of life benefits associated with SVR, published in English language from 2002–2013. For inclusion studies were required to enroll ≥100 patients and to report clinical endpoints including hepatocellular carcinoma, overall- or liver-related mortality, or progression of disease/complications (e.g. portal hypertension, esophageal varices). Review of economic studies on cost/cost-effectiveness of achieving SVR were focused on studies assessing boceprevir/telaprevir plus pegIFN and ribavirin as this represents the current standard of care in several jurisdictions worldwide. Quality of life evidence was required to use validated quality of life instruments and provide a quantitative analysis of the impact of SVR versus no treatment or treatment failure.

**Results:**

SVR is durable with late relapse rates over 4–5 year periods being in the range of 1–2%. Patients who achieve SVR frequently demonstrate some regression of fibrosis/cirrhosis and have a substantially reduced risk for hepatocellular carcinoma (relative risk [RR] 0.1–0.25), liver-related mortality (RR 0.03–0.2) and overall mortality (RR 0.1–0.3) in comparison with no treatment or treatment failure. In the 5 years post-treatment, medical costs for patients achieving SVR are 13-fold lower than patients not achieving SVR. Patients who achieve SVR also have health state utility values that are 0.05 to 0.31 higher than non-responders to treatment.

**Conclusions:**

SVR represents the fundamental goal of antiviral treatment for patients infected with chronic HCV, so as to reduce risk of liver disease progression. Achievement of SVR has implications beyond those of clearing viral infection; it is associated with improved long-term clinical outcomes, economic benefits and improved health-related quality of life.

**Electronic supplementary material:**

The online version of this article (doi:10.1186/s12879-015-0748-8) contains supplementary material, which is available to authorized users.

## Background

On a global level over 2% of the population are estimated to be infected with the hepatitis C virus (HCV), which corresponds to a prevalent population of >180 million people with chronic infection [1]. For many patients who become chronically infected, HCV causes slow, progressive damage to the liver and represents one of the leading causes of cirrhosis and hepatocellular carcinoma (HCC) [2]. Moreover, the slow insidious nature of disease progression means that many patients are unaware of their status until the later stages of disease.

Six major genotypes of HCV exist in many regions and the current standard of care for patients with HCV genotype 1 is therapy with a direct acting antiviral (DAA) in combination with ribavirin alone or combined with pegIFN or a combination of two DAAs (with or without ribavirin). The present study focuses on HCV genotype 1 as this genotype occurs in all regions and is the predominant genotype in many regions.

The effectiveness of antiviral treatment, the extent to which treatment can clear viral infection is assessed according to the proportion of patients achieving sustained virologic response (SVR). SVR is the fundamental goal of treatment and is defined as undetectable (or below the lower limit of quantification) HCV RNA at 12–24 weeks after cessation of treatment [3,4]. SVR rates with a DAA in combination with pegIFN plus ribavirin (PR) currently range from approx. 80–90% for treatment-naïve patients [5-8], whilst SVR rates of up to 99% have been reported with combinations of two DAAs [9]. Similarly, SVR rates of up to 99% have been reported in treatment-experienced (non-responders and relapsers) patients treated with two-DAA combinations [10].

Although considered a surrogate endpoint (a biomarker indicative of viral clearance rather than a finite endpoint such as presence/absence of disease or mortality), SVR is widely accepted as the best available indicator of viral clearance and a subject with SVR is generally considered cured [11]. Rates of late relapse are extremely low and long-term (up to 4 years) studies of patients treated with pegIFN have shown that SVR is durable, with approximately 99% of patients remaining virus-free, although the patient is still at risk of subsequent reinfection [12].

To date, the vast majority of clinical trials in HCV, including phase III trials of boceprevir and telaprevir, have used SVR at 24 weeks after the planned end of treatment (SVR24) as the primary endpoint. However, research in the field of HCV is currently advancing at a rapid pace and SVR 12 weeks after the end of treatment is now used as the primary endpoint in most clinical studies. The concordance between SVR12 and SVR24 rates has been investigated, and a high level of concordance was observed, suggesting that SVR12 represents a valid clinical endpoint [13,14]. Specifically, analysis was performed by the FDA in which data from fifteen Phase 2 and 3 trials (n = 12,000 patients) were combined to assess the concordance between SVR24 and SVR12. This analysis showed that concordance was observed between SVR12 and SVR24 for all treatments: 98% of patients with SVR212 had SVR24 [15].

As mentioned, SVR is the most commonly used endpoint in clinical trials in hepatitis C because the use of incidence of HCC or liver-related mortality as an endpoint is impractical within the context of a clinical trial. Patients with SVR following 24–48 weeks of treatment are generally considered to be permanently cured. While long-term follow-up is still required to fully assess the impact of SVR on hard clinical endpoints such as the progression to compensated or decompensated cirrhosis, HCC and liver-related mortality, it has been shown that patients who achieve SVR have a considerably reduced incidence of liver-related complications in comparison with those who fail treatment. As well as clinical implications, SVR rates can be anticipated to have an impact on the economic burden and humanistic burden of disease. HCV-related complications, such as HCC or liver-transplantation are associated with high direct medical costs and high levels of healthcare resource utilization [16], therefore any reduction in the incidence of HCV-related complications may have a considerable long-term economic benefit. This also extends to work productivity, as patients with SVR have higher post-treatment employment rates than those who fail treatment [17,18]. However, in the short-term improvements in SVR rates may be associated with increased pharmacy costs; for example, in an analysis in the French setting Deuffic-Burban *et al*. projected that the introduction of triple therapy would lead to a 3–4 fold increase in the number of genotype 1 patients receiving treatment at a cost of EUR 497–638 million [19]. As HCV is a transmissible disease, from a public health perspective, benefits of improved SVR rates include a reduced prevalent population and therefore the potential for lower transmission and incidence rates.

The objectives of the current study were to perform a literature review to understand the link between the clinical implications of achievement of SVR with the economic and patient quality of life implications by, firstly, exploring the clinical validity of SVR as an endpoint in terms of the impact of SVR on the incidence of liver-related complications including mortality and HCC and secondly, to assess the impact of attainment of SVR in terms of long-term economic outcomes and quality of life in patients infected with chronic HCV infection.

## Methods

The search strategy for the literature review was designed using high level Medical Subject Heading (MeSH) terms and supplemented with free text terms and adapted for the PubMed, EMBASE and Cochrane Library databases as required; all initial searches were run on 08 January 2013 (subsequent searches with the same search terms were run on 29 April 2014 to capture studies published since the initial review was performed). For the PubMed searches, MeSH terms used included Hepatitis C [MeSH] OR Hepacivirus [MeSH]; free-text terms were used to identify articles focusing on sustained virologic response (wildcards were used to capture variations in terminology). For the EMBASE searches, MeSH terms were mapped to EMBASE equivalents using the “map term” functionality.

The review was limited to articles published in the last 10 years and for inclusion, studies were required to be published in English and have a minimum enrollment of 100 patients (Table 1) (a minimum cohort size of 100 patients was chosen to focus on relatively large scale studies that could detect relatively small differences in outcomes and to preclude small scale pilot studies conducted in highly selective patient populations). The focus of the review was on patients with HCV genotype 1, and studies exclusively in patients with HCV genotypes 2 and 3, or in patients with HIV coinfection, were excluded. Clinical studies were also required to have a minimum follow up of 1-year post-cessation of treatment, compare outcomes in patients with SVR versus either untreated patients or those failing to achieve SVR, and report hard clinical endpoints; studies reporting biochemical parameters only, such as alanine amino transferase levels, were excluded. Cost-effectiveness studies were also limited to studies incorporating analyses of protease inhibitor-based triple therapy regimens; studies evaluating pegIFN plus ribavirin in comparison with pegIFN or IFN alone or no treatment were excluded. Studies reporting on health-related quality of life evidence were required to use validated quality of life instruments and provide a quantitative analysis of the impact of SVR versus no treatment or treatment failure.

**Table 1:** **Summary of inclusion and exclusion criteria applied in the literature review**

**Inclusion criteria**	**Exclusion criteria**
**All studies**	All studies
Published 2003–2014	Conducted exclusively in HIV co-infected patients
Published in English	Conducted exclusively in pediatric patients
Conducted in patients with chronic HCV	Wrong publication type: letters, case studies, editorials and commentaries were excluded
	Conducted exclusively in patients with genotypes 2, 3, 4, 5 or 6
**Clinical studies**	Clinical studies
Minimum 1 year post-treatment follow-up	<100 patients
Report hard clinical endpoints (e.g. overall mortality, incidence of hepatocellular carcinoma)	Endpoints limited to biochemical parameters only (e.g. aminotransferase levels only)
**Health economic studies**	**Health economic studies**
Assessing cost-effectiveness of protease inhibitors versus pegIFN plus ribavirin, pegIFN, IFN or no treatment	Assessing pegIFN plus ribavirin versus pegIFN, IFN or no treatment
**Quality of life studies**	**Quality of life studies**
Presentation of quantitative results using a validated quality of life instrument	

HCV, hepatitis C virus; HIV; human immunodeficiency virus; pegIFN, pegylated interferon; IFN, interferon.

**Figure 1:**
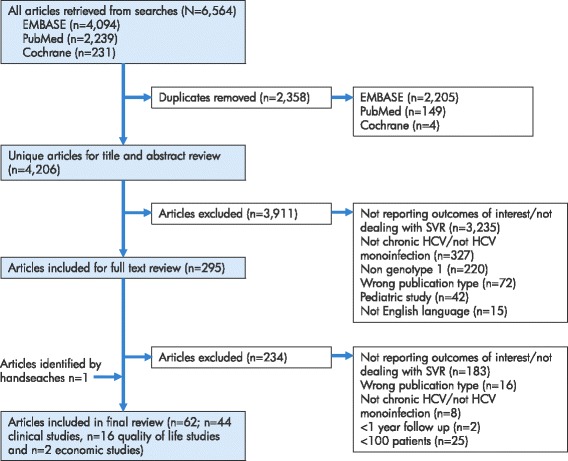
**Diagram of literature review process.** Note: the original literature searches were re-run in April 2014 to capture publications published since the original searches. A total of twenty additional clinical articles and three additional economic studies were identified.

The literature searches across the three databases identified a total of 4,206 unique hits after two rounds of screening (first round screening by title and abstract only and second round full-text screening of short-listed articles.) A total of 44 clinical studies (including 4 meta-analyses), 15 quality of life studies (one additional quality of life was identified in supplementary hand searches) and 2 economic studies were included in the final analysis (Figure 1). The review was performed in line with PRISMA guidance and a schematic diagram of the literature review process is shown in the Additional file 1. Updated searches performed in April 2014 identified an additional 20 clinical studies and 3 cost/cost-effectiveness studies.

## Results

### Clinical benefits

The literature review process identified a large number of studies that examined the impact of SVR on the long-term risk of a number of clinical outcomes including incidence of HCC, liver transplantation, liver-related mortality and overall mortality in populations with differing levels of severity. Data were captured from a large range of patient populations in terms of relative prevalence of different HCV genotypes, severity of liver disease at baseline and treatment type.

### Hepatocellular carcinoma

On a global level, HCV is one of the leading causes of HCC, and is typically associated with a poor prognosis. A total of 34 studies [20-48] including five meta-analyses [49-53] that examined the impact of SVR on risk of HCC were identified (Table 2). The overwhelming consensus of the results of the studies included was that patients who achieve SVR have a considerably reduced risk for HCC in comparison with untreated patients or those who fail to achieve SVR. However, the magnitude of this effect varied, with reported RRs for HCC in patients with SVR versus non-responders or untreated patients ranging from 0.09–0.35.

**Table 2:** **Summary of clinical studies reporting the impact of SVR on HCC**

**Study**	**Setting**	**Sample size**	**Mean follow up**	**Study details**	**Key findings**
Coverdale *et al*. 2004 [47]	Australia	455	9 years^a^	Retrospective cohort study including 384 treated with IFN alone, (n = 71 untreated) including patients with cirrhosis	Overall 9-year incidence of HCC was 10% for untreated, 11% for non-response and 2% for SVR
Van der Meer *et al*. 2013 [20]	Europe and Canada	248	8.3 years^a^	Cohort of consecutive genotype 1 patients with advanced fibrosis, 24% with SVR	HR (95% CI) for HCC for SVR versus non-SVR was 0.20 (0.06–0.69) (p = 0.011)
Van der Meer *et al*. 2012 [38]	Europe and Canada	530	8.4 years^a^	Retrospective cohort study in patients with advanced fibrosis/cirrhosis treated with IFN, IFN plus ribavirin or pegIFN plus ribavirin, median follow up 8.4 years, 68% genotype 1	Rate (per 100 patient years) for HCC were 0.55 (0.14–0.96) for SVR vs. 2.63 (1.83–3.82) without SVR (p < 0.001)
Braks *et al*. 2007 [43]	France	113	8.2 (3.1) years	Retrospective cohort study in patients with compensated cirrhosis treated with IFN or pegIFN-based treatment	Proportion of patients with HCC was 2.7% for SVR versus 31.6% for non-SVR
Cardoso *et al*. 2010 [42]	France	307	3.5 years^a^	Retrospective analysis in patients with bridging fibrosis or cirrhosis treated with IFN, pegIFN or pegIFN plus ribavirin	Adjusted HR (95% CI) for non-SVR versus SVR was 3.06 (1.12–8.39) (p = 0.029) for HCC
Bruno *et al*. 2007 [44]	Italy	883	96.1 months	Retrospective database analysis in patients treated with IFN monotherapy with no cirrhosis or decompensation, 73.5% genotype 1	Adjusted HR (95% CI) for non-SVR versus SVR was 2.59 (1.13–5.97) (p = 0.025) for HCC
Calvaruso *et al*. 2013 [23]	Italy	444	69 months^a^ (range 24–130 months)	Prospective cohort study in PR-treated patients with compensated cirrhosis, 83% genotype 1, 24% with SVR	HR (95% CI) for HCC for non SVR versus SVR = 4.44 (1.30–15.11) (p = 0.017)
Pellicelli *et al*. 2013 [27]	Italy	172	5 years^a^	Retrospective-prospective study in patients with HCV genotype 1 treated with pegIFN plus ribavirin, 34% with cirrhosis	Multivariate OR (95% CI) for development of HCC for no SVR versus SVR = 3.58 (0.9–14.3) (p = 0.06)
Hara *et al*. 2014 [24]	Japan	1,125	Not stated	Retrospective cohort study in PR-treated (SVR and non SVR) and untreated patients	HR (95% CI) for HCC for SVR versus non-SVR and untreated = 0.12 (0.03–0.48) (p = 0.003)
Ikeda *et al*. 2006 [35]	Japan	2,166	15 years	Retrospective cohort study in patients with HCV patients (n = 512 untreated, n = 1,654 treated with IFN-based therapy)	Crude rate of HCC at 15 years was 13.9% for all treated patients, 23.9% for untreated and 7.5% for SVR
Imai *et al*. 2010 [28]	Japan	568	11 years	Retrospective cohort study in consecutive HCV patients treated with IFN monotherapy	HR (95% CI) for HCC for SVR versus non-treated patients was 0.20 (0.08–0.50) (p < 0.001) for patients <60 years and 0.23 (0.08–0.64) (p = 0.005) for patients >60 years
Imazeki *et al*. 2005 [46]	Japan	459	8.9 (3.2) years	Retrospective cohort study in patients, inc patients with cirrhosis, treated with IFN alone (n = 355) or untreated (n = 104), n = 116 patients achieved SVR	In the total population, annual incidence of HCC was 0.5% for SVR versus 2.6% for non-responders; corresponding figures for patients with cirrhosis were 9% and 34%, respectively
Kobayashi *et al*. 2007 [29]	Japan	1,124	66 months^a^ (range 12–197 months)	Retrospective cohort study in HCV patients treated with IFN or IFN plus ribavirin (373 with SVR, 751 without SVR)	HCC developed in 3.5% SVR patients versus 8.1% non-SVR patients. SVR HCC patients had a significantly more advanced stage of fibrosis (p < 0.001)
Maruoka *et al*. 2012 [40]	Japan	721	9.9 (5.3) years	Retrospective cohort study in patients treated with monotherapy (n = 577, of which n = 221 (38.3%) achieved SVR and n = 144 untreated patients	Annual rate of HCC development was 2.71% for untreated patients, 2.31% for non-SVR and 0.24% for SVR (p < 0.0001)
Moriyama *et al*. 2005 [31]	Japan	269	>6 years	Retrospective study in patients with cirrhosis treated with IFN-based treatment	Mean annual incidence of HCC was 0.78% for SVR versus 0.17% for non-responders with ALT <80 IU and 4.68% for ALT >80 IU
Ogawa *et al*. 2013 [25]	Japan	1,013	3.6 years^a^	Prospective multicenter study in patients treated with pegIFN plus ribavirin, 70.1% had HCV genotype 1 and 14.8% had cirrhosis at baseline	HR (95% CI) for HCC relative to SVR = 1.50 (0.65–3.44) (p = 0.34) for relapse and breakthrough and 3.72 (1.69–8.18) (p = 0.001) for non-response
Ogawa *et al*. 2012 [37]	Japan	1,015	3.8 years (2–6 years)	Prospective multicenter study in patients treated with pegIFN plus ribavirin (n = 712 genotype 1, n = 303 genotype 2)	6-year cumulative incidence of HCC was 3.4% for SVR versus 21.2% for non-response group (p < 0.0001) and 6.4% for transient response (ns)
Sasaki *et al*. 2014 [22]	Japan	916	Not stated	Retrospective study of IFN-treated patients	Incidence of HCC was 3.6% in patients who achieved SVR vs. 21.2% in non-SVR patients
Sasaki *et al*. 2011 [34]	Japan	236	50 months^a^	N = 236 patients with IFN-based treatment, median follow up 50 months	No significant difference in incidence of HCC for SVR versus non-SVR
Watanabe *et al*. 2011 [32]	Japan	1,865	4.25 years^a^	Retrospective cohort study in patients treated with pegIFN plus ribavirin, n = 999 (54%) with SVR	5 year cumulative incidence of HCC was 1.1% in patients with SVR and 7.1% in non-SVR patients (p < 0.001)
Yoshida *et al*. 2004 [36]	Japan	2,787	>6.5 years^a^	Retrospective database analysis in HCV patients (n = 395 untreated, n = 836 SVR, and n = 1,556 non-SVR)	HR (95% CI) for HCC for non-SVR versus no treatment was 0.835 (0.625–1.125) (p = ns).
					Annual incidence of HCC in SVR was 0.05–0.40% for F0–F1 and 0.15–3.20% for F4. For non-SVR annual incidence was 0.05–1.03% for F0–F1 and 0.29–12.5% for F4 (depending on age and gender)
Velosa *et al*. 2011 [39]	Portugal	130	6.4 (4.0) years	Retrospective cohort study in patients with cirrhosis treated with IFN, IFN plus ribavirin or pegIFN plus ribavirin	HR (95% CI) for HCC for SVR versus non-SVR was 0.09 (0.01–0.77) (p = 0.024)
Aleman *et al*. 2013 [26]	Sweden	351	5.3 years	Prospective multicenter study in patients with HCV-related cirrhosis treated with pegIFN plus ribavirin, 50% genotype 1	HR (95% CI) for HCC for SVR versus non-SVR = 0.38 (0.14–0.88) (p = 0.04)
Hung *et al*. 2006 [30]	Taiwan	132	37 months^a^ (12–63 months)	Retrospective cohort study in HCV patients with cirrhosis , inc. patients with HBV or HIV coinfection, 56% genotype 1b, treated with pegIFN plus ribavirin	4 year cumulative incidence of HCC was 28% in non-SVR versus 8% in SVR group (p = 0.0178)
Shih *et al*. 2012 [48]	Taiwan	3,988	34.6 months^a^	Retrospective analysis of patients with HCV monoinfection, (n = 344 patients treated with IFN-based treatment, n = 216 with SVR)	Adjusted HR (95%CI) for SVR versus untreated was 0.23 (0.06–0.94) (p = 0.041) for HCC
Wang *et al*. 2011 [33]	Taiwan	164	8 years	Retrospective cohort study in patients treated with pegIFN plus ribavirin	Incidence of HCC was 8.8% for patients with an SVR versus 14.3% for untreated patients (p = 0.352)
Yu *et al*. 2006 [45]	Taiwan	1,619	5.2 years	Prospective study in patients with or without cirrhosis (n = 562 untreated and n = 1,057 treated with IFN or IFN plus ribavirin)	RR (95% CI) for HCC versus untreated was 0.245 (0.13–0.46) (p < 0.0001) for SVR and 0.990 (0.635–1.541) (p = 0.963) for non-SVR
Morgan *et al*. 2010 [41]	United States	140	78.6 (15.9) months	Prospective analysis from the HALT-C trial in patients with advanced fibrosis treated with pegIFN plus ribavirin and achieving SVR	HR (95% CI) for SVR versus non response was 0.19 (0.04–0.80) for HCC
Wang *et al.* 2013 [21]	Not stated	138	8 years	Patients (mean age 56 years) treated with PR, 80% achieved SVR	8-year incidence of HCC was 13.5% for SVR patients, 23.5% for relapsers and 20% for non-responders (p = 0.518)

^a^Median follow up.

ALT, alanine aminotransferase; CI, confidence interval; HCC, hepatocellular carcinoma; HR, hazard ratio; IFN, interferon; ns, not significant; SVR, sustained virologic response.

The 2010 meta-analysis by Singal *et al*. showed that patients who had SVR (following treatment with IFN alone or IFN plus ribavirin) had a RR (95% CI) for HCC of 0.35 (0.26–0.46) in comparison with non-responders [49]. Similarly, the meta-analysis by Kimer *et al*. reported a RR (95% CI) for HCC of 0.15 (0.05–0.45); however, the comparator group was untreated patients, rather than non-responders to therapy [51]. Notably, the analysis by Singal *et al*. included only studies in patients with cirrhosis, whereas the analysis by Kimer *et al*. included two studies in mixed or non-cirrhotic patients.

Studies examining the impact of SVR on risk for HCC in Japan are of particular interest owing to the high relative prevalence of HCV genotype 1b, (which is associated with a higher incidence of HCC than genotype 1a) and high incidence of HCV-associated HCC in this setting. For HCV patients with cirrhosis the annual probability of HCC is 1–4%, although this increases to 5–8% for patients with HCV genotype 1b [54,55]. Japan-based studies also showed that SVR was associated with reduced risk for HCC versus non-response, although as expected the absolute risk in both SVR and non-SVR population was increased with advanced age and increased severity of fibrosis. For example, Yoshida *et al*. determined SVR-related gain in HCC-free survival as both a function of age and fibrosis level (as measured by METAVIR F0–F4) [36]. For male patients with F0/F1 stage disease the gain in HCC-free survival with SVR was 2.48 years for patients aged 30 years, reducing to 0.15 years for patients aged 80 years. For patients with F4 stage disease SVR-induced gain in HCC free survival was 15.98 years at age 30 years, but only 2.38 years at age 80 years [36]. In another Japanese study by Imazeki *et al*., in the overall treated HCV population they report an annual HCC incidence of 0.5% for those with SVR versus 2.6%; whereas in patients with cirrhosis, the corresponding figures were 1.4% and 5.9%, respectively [46]. Similar findings were reported in other studies in the Japanese setting [22,24,29]. Only two studies (out of thirteen) from the Japanese setting reported no difference in the incidence of HCC for patients achieving SVR versus those without SVR [34].

### Liver-related mortality

Analysis of clinical studies also showed that patients who achieve SVR have a substantially lower risk of liver-related mortality and overall mortality than non-responders to treatment, irrespective of genotype, setting or disease severity level, with a considerable proportion of studies showing that this reduction in risk was statistically significant (Table 3). In individual studies the RR for overall mortality for patients with SVR versus non-response or no treatment ranged from 0.14–0.70, whilst the corresponding figures for liver-related mortality were 0.03–0.22. As with HCC studies, the magnitude of the effect of SVR on mortality risk varied considerably between studies, which may be attributable in part to differences in patient characteristics such as mean age and disease stage prior to treatment. A 2010 meta-analysis reported a RR (95% CI) for liver-related mortality of 0.23 (0.10–0.52) for SVR patients compared with treatment failures, although if only patients with advanced fibrosis/cirrhosis were included this figure decreased to 0.13 (0.06–0.29) [50]. These findings were echoed in individual studies. For example, a large scale (N = 1,215 treatment-naïve patients), UK-based retrospective study reported a multivariate HR (95% CI) for liver-related death for SVR patients of 0.22 (0.09–0.58) (p < 0.01) [56]. Similarly, an Italian study of HCV patients (with no cirrhosis) reported that not achieving SVR (versus SVR) increased the HR (95% CI) for liver-related death to 6.97 (1.70–28.42) [44]. Additionally, studies in the Japanese setting reported similar findings, with two studies reporting RRs for liver-related mortality of 0.03–0.04 for patients achieving SVR versus untreated patients [57,58].

**Table 3:** **Summary of clinical studies reporting the impact of SVR on all-cause and liver-related mortality**

**Study**	**Setting**	**Sample size**	**Mean follow up**	**Study details**	**Outcomes assessed**	**Key findings**
Selzner *et al*. 2009 [59]	Canada	446	68 months^a^	Retrospective cohort study in liver transplant recipients treated with IFN-based therapy	Overall survival	Actuarial 5-year survival rates were 96% for SVR and 69% for non-response (p < 0.0001)
Tanaka *et al*. 2013 [60]	Canada	245	5.7 years	Retrospective single center study in liver transplant recipients undergoing treatment (agents not stated)	All cause mortality	HR for all cause mortality for SVR versus non-response = 0.091 (0.04–0.21) (p < 0.001). HR for all cause mortality for relapse versus non-response = 0.19 (0.06–0.63) (p = 0.006)
Van der Meer *et al*. 2012 [38]	Europe and Canada	530	8.4 years	Retrospective cohort study in patients with advanced fibrosis/cirrhosis treated with IFN, IFN plus ribavirin or pegIFN plus ribavirin, 68% genotype 1	All cause mortality	HR for all cause mortality for SVR versus non-SVR was 0.25–0.26 (p < 0.001)
Van der Meer *et al*. 2012 [61]	Europe and Canada	248	8.3 years^a^	Retrospective cohort study in patients with HCV genotype 1 with cirrhosis, treated with IFN-based treatment, 88% treatment-naïve at baseline	All cause mortality	Unadjusted HR (95% CI) for all cause mortality for SVR 0.20 (0.06–0.64) (p = 0.007)
Aguilera *et al*. 2012 [62]	France	114	Not stated	liver transplant recipients treated with pegIFN plus ribavirin	Overall survival	For patients with F0–F1, 10 year survival was 100% for SVR versus 76% for non-response (p = 0.024). For patients with F3–F4, 7-year survival was 85% for SVR versus 72% for non-response (p = ns)
Cardoso *et al*. 2010 [42]	France	307	3.5 years^a^	Retrospective analysis in patients with bridging fibrosis or cirrhosis treated with IFN, pegIFN or pegIFN plus ribavirin	Liver-related mortality	Adjusted HR (95% CI) for non-SVR versus SVR was 3.71 (1.05–13.05) (p = 0.041) for liver-related mortality
Kutala *et al*. 2013 [63]	France	484	4.5 years^a^	Retrospective study in patients with advanced fibrosis, SVR rate was 30% in treated patients	All cause mortality	5 year survival rate was 100% in those with SVR vs. 54% for those without SVR (p < 0.0001), HR (95% CI) for mortality for non-SVR versus SVR was 6.8 (2.5–20.5)
Bruno *et al*. 2007 [44]	Italy	883	96.1 months	Retrospective database analysis in patients treated with IFN monotherapy with no cirrhosis or decompensation, 73.5% genotype 1	Liver-related mortality	Adjusted HR (95% CI) for non-SVR versus SVR was 6.97 (1.70–28.42) (p = 0.0007) for liver-related mortality
Calvaruso *et al*. 2013 [23]	Italy	444	69 months^a^ (range 24–130 months)	Prospective cohort study in PR-treated patients with compensated cirrhosis, 83% genotype 1, 24% with SVR	Liver-related mortality	HR (95% CI) for liver related death for no SVR versus SVR = 6.56 (2.06–20.92) (p = 0.001)
Hara *et al.* 2014 [24]	Japan	1,125	Not stated	Retrospective cohort study in PR-treated (SVR and non SVR) and untreated patients	All cause mortality	HR (95% CI) for all cause mortality for SVR vs non-SVR and untreated = 0.08 (0.01–0.55) (p = 0.011)
Imazeki *et al*. 2003 [58]	Japan	459	8.2 (2.9) years	Retrospective cohort study in consecutive patients with CHC (335 treated with IFN and 104 untreated)	All-cause mortality, liver-related mortality	Adjusted RR (95%CI) for all cause mortality for SVR versus untreated was 0.22 (0.07–0.71) (p = 0.0114). Adjusted RR (95% CI) for liver-related death was 0.03 (0.003–0.28) (p = 0.0017)
Kasahara *et al*. 2004 [57]	Japan	2,954	6.0 (2.2) years	Retrospective cohort study in HCV patients with stage F0–F4 fibrosis, (n = 2,698 treated with IFN alone, n = 256 untreated)	All cause mortality, liver-related mortality	RR (95% CI) for all cause mortality versus untreated was 0.14 (0.06–0.35) (p < 0.001) for SVR and 0.78 (0.43–1.39) (p = 0.394) for non-response. RR (95% CI) for liver-related mortality versus no treatment was 0.04 (0.01–0.30) (p = 0.002) for SVR and 1.02 (0.54–1.90) (p = 0.962)
Maruoka *et al*. 2012 [40]	Japan	721	9.9 (5.3) years	Retrospective cohort study in patients treated with monotherapy (n = 577, of which n = 221 (38.3%) achieved SVR and n = 144 untreated patients	Overall mortality, liver-related mortality	Annual liver-related mortality rate was 2.52% for untreated patients, 1.26% for non-SVR and 0.1% for SVR. Multivariate HR for all cause mortality versus untreated was 0.84 (0.50–1.42) for non-SVR and 0.17 (0.08–0.40) for SVR
Uenishi *et al*. 2008 [64]	Japan	209	4.1 years^a^	Retrospective cohort study in patients who underwent curative surgery for early stage HCC (n = 139 had no antiviral treatment, remainder treated with pegIFN plus ribavirin)	Tumor-free survival and recurrence of HCC	Tumor-free survival rate at 5 years was 54% for SVR group versus 23% for non-SVR/untreated group (p < 0.001)
Velosa *et al*. 2011 [39]	Portugal	130	6.4 (4.0) years	Retrospective cohort study in patients with cirrhosis treated with IFN, IFN plus ribavirin or pegIFN plus ribavirin	Liver-related mortality	Liver-related mortality rate during follow up was 21% for non-SVR versus 0% for SVR
Aleman *et al*. 2013 [26]	Sweden	351	5.3 years	Prospective multicenter study in patients with HCV-related cirrhosis treated with pegIFN plus ribavirin, 50% genotype 1	All cause mortality, liver-related mortality	HR (95% CI) for liver-related mortality for SVR versus non-SVR = 0.18 (0.05–0.45) (p = 0.001)
						HR (95% CI) all cause mortality for SVR versus non SVR = 0.36 (0.18–0.68) (p = 0.003)
Shih *et al*. 2012 [48]	Taiwan	3,988	57.7 months^a^	Retrospective analysis of patients with HCV monoinfection, (n = 344 patients treated with IFN-based treatment, n = 216 with SVR)	Liver-related mortality	Adjusted HR (95%CI) for SVR versus untreated was 0.19 (0.05–0.77) (p = 0.02) for liver-related mortality
Yu *et al*. 2006 [45]	Taiwan	1,619	5.2 years	Prospective study in patients with or without cirrhosis (n = 562 untreated and n = 1,057 treated with IFN or IFN plus ribavirin)	Overall morality	RR (95% CI) for overall mortality versus untreated control was 0.37 (0.14–0.99) (p = 0.047) for SVR and 1.32 (0.57–3.07) (p = 0.524)
Innes *et al*. 2012 [56]	United Kingdom	1,215	5.3 years	Retrospective cohort study in previously naïve patients, 36% genotype 1, treated with IFN-based therapy, 14% patients with cirrhosis at baseline	Liver-related mortality	Adjusted HR (95% CI) for SVR versus non-SVR was 0.22 (0.09–0.58) for liver-related mortality (p < 0.01)
Backus *et al*. 2011 [65]	United States	22,942	3.8 years^a^	Retrospective database analysis in n = 12,166 genotype 1, n = 2,904 genotype 2, and 1,794 genotype 3 patients treated with pegIFN plus ribavirin	All cause mortality	Adjusted HR (95% CI) for all cause mortality for SVR versus non SVR in HCV genotype 1 was 0.70 (0.59–0.83) (p < 0.0001)
Cozen *et al*. 2013 [66]	United States	358	10 years	Retrospective database analysis in patients with HCV treated with IFN monotherapy or pegIFN plus ribavirin, 69% genotype 1 and 7.3% with cirrhosis at baseline	All cause mortality	HR (95% CI) for death or liver transplant vs. never treated patients = 0.23 (0.07–0.75) for SVR and 0.56 (0.24–1.32) for non-responder
Dieperink *et al.* 2014 [67]	United States	536	7.5 years^a^	Retrospective chart review of treated patients, 70% genotype 1, SVR rate of 41%	All cause mortality, liver-related mortality or transplant	HR (95% CI) for all cause mortality for SVR vs. non-SVR = 0.47 (0.26–0.85) (p < 0.012)
						HR (95% CI) for liver-related death or transplant for SVR vs. non-SVR = 0.23 (0.08–0.66) (p = 0.007)
Morgan *et al*. 2010 [41]	United States	140	78.6 (15.9) months	Prospective analysis from the HALT-C trial in patients with advanced fibrosis treated with pegIFN plus ribavirin and achieving SVR	All cause mortality, liver-related mortality, liver transplantation	HR (95% CI) for SVR versus non response was 0.17 (0.06–0.46) for all cause mortality or transplant and 0.12 (0.03–0.48) for liver-related mortality or transplant
Singal *et al*. 2013 [68]	United States	242	5 years	Retrospective single center study in patients treated with pegIFN plus ribavirin, 68% genotype 1, 31% with histological cirrhosis	All cause mortality	HR for mortality for SVR versus non-response = 0.11 (0.03–0.47)

^a^Median follow up.

ALT, alanine aminotransferase; CI, confidence interval; HCC, hepatocellular carcinoma; HR, hazard ratio; IFN, interferon; ns, not significant; SVR, sustained virologic response.

The benefits of SVR in terms of reduced risk for liver-related mortality were apparent regardless of baseline severity. A multicenter study by van der Meer *et al*. [38] with over 8 years of follow up was conducted exclusively in patients with advanced fibrosis or cirrhosis at baseline. Van der Meer showed that SVR led to a 3-fold reduction in the overall mortality rate (1.01 [0.46–1.56] per 100 patient years for SVR versus 2.93 [2.36–3.51] per 100 patient years for those without SVR; p < 0.001) and a 30-fold reduction in liver-related mortality or transplant (0.23 [0.01–0.50] per 100 patient years for SVR versus 3.20 [2.58–3.82] per 100 patient years for those without SVR; p < 0.001) [38].

### Overall mortality

Achievement of SVR has also been shown to reduce the risk of overall mortality (Table 3). For example, in a US-based study, SVR was associated with a HR (95% CI) versus non SVR for all-cause mortality (for genotype 1 only) was 0.70 (0.59–0.83) (p < 0.0001) [65]. Other studies report a much lower figure, with Morgan *et al*. reporting a HR (95% CI) for all-cause mortality or liver transplant of 0.17 (0.06–0.46) [41].

### Other complications

Four studies identified in the review (conducted in Japan, Spain and the United States), showed that patients with SVR had a reduced risk for new onset diabetes in comparison with those not achieving SVR; in patients who achieved SVR the risk of developing diabetes was approximately 2-fold lower than for patients who failed treatment (Table 4). In all four studies investigating this associated the reduced risk for type 2 diabetes with SVR was statistically significant [69-72].

**Table 4:** **Summary of clinical studies reporting the impact of SVR on liver-related complications**

**Study**	**Setting**	**Sample size**	**Mean follow-up**	**Study details**	**Outcomes assessed**	**Key findings**
Coverdale *et al*. 2004 [47]	Australia	455	9 years^a^	Retrospective cohort study including 384 treated with IFN alone, (n = 71 untreated) including patients with cirrhosis	Liver-related complications	Overall 9-year incidence liver-related complication rate was 25% for untreated, 25% for non-response and 2% for SVR
Abergel *et al*. 2004 [73]	France	163	Not stated	Retrospective cohort study in patients with severe fibrosis treated with IFN alone (n = 64) or IFN plus ribavirin (n = 99)	Progression of fibrosis	Fibrosis progression rate decreased in both responders and non-responders to treatment. 33% SVR regressed from cirrhosis to severe fibrosis; corresponding figure for non-responders was 9% (p = 0.058)
Braks *et al*. 2007 [43]	France	113	8.2 (3.1) years	Retrospective cohort study in patients with compensated cirrhosis treated with IFN, IFN plus ribavirin or pegIFN plus ribavirin	Liver-related complications	Proportion of patients with ascites was 5.4% for SVR vs. 10.5% for non-SVR, rates of digestive hemorrhage were 2.7% vs. 5.3%, respectively
Poynard *et al*. 2013 [74]	France	933	6.3 years	Prospective cohort study in HCV patients, 62% genotype 1	Regression of fibrosis, progression to cirrhosis	HR (95% CI) for regression of fibrosis at 10 years for SVR versus non-response = 4.94 (2.59–9.44) (p < 0.001)
						HR (95% CI) for progression to cirrhosis = 0.185 (0.106–0.264) for SVR and 0.173 (0.123–0.224)
Roche *et al*. 2008 [75]	France	113	31.4 months^a^	Open label study in with liver transplant recipients treated with IFN plus ribavirin or pegIFN plus ribavirin, 75% genotype 1	Fibrosis stage	For SVR mean (SD) necroinflammatory grade decreased from 1.9 (0.6) to 1.0 (0.6) post-therapy and improved in 71.5% and remained stable in 26% SVR patients; corresponding figures in non-SVR patients were 51.5% and 46%, respectively
Wiese *et al*. 2014 [76]	Germany	718	35 years	Prospective, community-based multicenter study in women with HCV genotype 1, SVR rate of 46% in treated patients	Cirrhosis	Incidence of cirrhosis at 35 years post-infection = 6.0% for SVR vs. 15.3% for non-SVR
Annicchiarico *et al*. 2012 [77]	Italy	135	44.4 months^a^	Prospective study in 135 HCV patients with cirrhosis	Portal hypertension	Development of portal hypertension was 10% for SVR versus 40% for non-SVR (p < 0.0005) progression of portal hypertension was 25% for SVR vs. 48% for non-SVR (p < 0.01)
Bruno *et al*. 2010 [78]	Italy	218	11.4 years^a^	Retrospective cohort study in patients with compensated cirrhosis (n = 149 patients treated with IFN or IFN plus ribavirin), but no esophageal varices	Esophageal varices	Esophageal varices developed in 32% untreated patients, 39% non-SVR patients and 0% SVR patients
D’ambrosio *et al*. 2011 [79]	Italy	127	77 months	Prospective cohort study in initially treatment-naïve patients with compensated cirrhosis, treated with IFN plus ribavirin	Esophageal varices (development of and or progression in size/severity)	Development/progression occurred in 5% SVR patients versus 15% non-SVR patients. 8-year cumulative probability of esophageal varices was 6% for SVR vs. 30% for non-SVR (p = 0.03)
Arase *et al*. 2009 [69]	Japan	2,842	6.4 years^a^	Retrospective cohort study in patients treated with IFN or pegIFN plus ribavirin, 6% patients had cirrhosis at baseline	Onset of type 2 diabetes	Adjusted HR (95% CI) for the development of diabetes for non-SVR vs. SVR was 2.73 (1.77–4.20) (p < 0.001)
Imazeki *et al*. 2005 [46]	Japan	459	8.9 (3.2) years	Retrospective cohort study in patients, inc patients with cirrhosis, treated with IFN alone (n = 355) or untreated (n = 104), n = 116 patients achieved SVR	Hepatic failure	In the total population, annual incidence of hepatic failure was 0% for SVR and 0.5% for non-responders; corresponding figures for patients with cirrhosis were 0% and 1.0%, respectively
Uenishi *et al*. 2008 [64]	Japan	209	4.1 years^a^	Retrospective cohort study in patients who underwent curative surgery for early stage HCC (n = 139 had no antiviral treatment, remainder treated with pegIFN plus ribavirin)	Tumor-free survival and recurrence of HCC	Tumor-free survival rate at 5 years was 54% for SVR group vs. 23% for non-SVR/untreated group (p < 0.001)
Lee *et al*. 2013 [80]	South Korea	315	45 months^a^	Retrospective chart review, 86% patients treated, 15% with cirrhosis at baseline, SVR rate of 75%	Cirrhosis	Cumulative 5 year rate of cirrhosis was 27.6% for patients without SVR vs. 0% for patients with SVR (p < 0.01)
Canete *et al*. 2013 [81]	Spain	105	9.3 years	Retrospective study of paired biopsy data in HCV patients with mild-moderate fibrosis treated with IFN plus ribavirin	Progression of fibrosis	Progression of fibrosis was reported in 5.3% patients with SVR and 50% patients with non-response (p < 0.0001). Fibrosis improved in 30.5% patients with SVR and 14.6% patients with non-response
Simo *et al*. 2006 [70]	Spain	234	5.7 years	Retrospective cohort study in patients with HCV (without severe fibrosis) treated with IFN or IFN plus ribavirin, 79% genotype 1	Onset of type 2 diabetes	HR (95% CI) for onset of diabetes for SVR vs. non-SVR was 0.48 (0.24–0.98) (p = 0.04)
Aleman *et al*. 2013 [26]	Sweden	351	5.3 years	Prospective multicenter study in patients with HCV-related cirrhosis treated with pegIFN plus ribavirin, 50% genotype 1	Decompensated cirrhosis (ascites, variceal bleeding, encephalopathy)	HR (95% CI) for hepatic decompensation for SVR vs. non SVR = 0.23 (0.08–0.53) (p = 0.002)
Innes *et al*. 2012 [56]	United Kingdom	1,215	5.3 years	Retrospective cohort study in previously naïve patients, 36% genotype 1, treated with IFN-based therapy, 14% patients with cirrhosis at baseline	Liver-related inpatient hospital episodes	Adjusted HRs (95% CI) for SVR versus non-SVR were 0.22 (0.15–0.34) for liver-related hospital episode (p < 0.01)
Cozen *et al*. 2013 [66]	United States	358	10 years	Retrospective database analysis in patients with HCV treated with IFN monotherapy or pegIFN plus ribavirin, 69% genotype 1 and 7.3% with cirrhosis at baseline	Cirrhosis	HR (95% CI) for development of cirrhosis vs. never treated = 0.68 (0.26–1.80) for SVR and 2.35 (1.18–4.69) for non-responders
Hyder *et al.* 2013 [72]	United States	20,486	5 years	Retrospective database analysis of US veterans with no history of diabetes treated between 1998–2007	Onset of type 2 diabetes	HR (95% CI) for onset of type 2 diabetes for SVR versus non-response was 0.76 (0.70–0.82) (p < 0.0001)
Oni *et al*. 2011 [71]	United States	8,687	>6 years	Retrospective database analysis in patients treated with pegIFN-based treatment	Onset of type 2 diabetes	Rate of new onset of diabetes was 10.2% for SVR group vs. 15% for non-SVR group

^a^Median follow up.

ALT, alanine aminotransferase; CI, confidence interval; HCC, hepatocellular carcinoma; HR, hazard ratio; IFN, interferon; ns = not significant; SVR, sustained virologic response.

### Economic implications

The incidence of late stage complications associated with HCV (e.g. HCC, decompensated cirrhosis and liver transplant) is a major contributor to the economic burden associated with HCV. In the US alone, direct annual costs associated with HCV exceed USD 1 billion [82], with annual per patient costs exceeding USD 50,000 for HCC and USD 110,000 for a single liver transplant [83]. Similarly, in Europe, a 5-country study by Vietri *et al*. showed that HCV patients have a high level of medical resource utilization leading to high direct costs as well as a high degree of absenteeism and presenteeism leading to high indirect costs. Indeed, Vietri *et al*. report direct annual costs of EUR 1,147 and indirect costs of EUR 7,533 per patient [84]. New antiviral treatment regimens that increase the SVR rate have the potential to influence future complication rates and therefore the overall economic burden; however, as triple therapy regimens are also associated with increased pharmacy costs in comparison with pegIFN plus ribavirin alone, cost-effectiveness analyses are required in order to quantify the estimated long-term clinical and economic benefits. The initial literature review and update captured a total of five studies that specifically assessed the economic benefits of treatment in terms of cost per SVR achieved or cost of SVR versus failure (Table 5) [85-89].

**Table 5:** **Summary of literature relating to the health economic implications of SVR**

**Study (setting)**	**Patients**	**Interventions**	**Key findings**
Morais *et al*. 2013 (Brazil) [86]	Treatment-naïve patients with genotype 1 with F2 fibrosis in Brazil	Boceprevir plus PR and telaprevir plus PR	In the public health system cost per SVR was BRL 50,751 for telaprevir plus PR and BRL 63,481 for boceprevir plus PR. In the private health system cost per SVR was BRL 88,508 for telaprevir plus PR and BRL 82,518 for boceprevir plus PR
Backx *et al*. 2014 (UK) [85]	Treated genotype 1 patients	Patients treated with PR for a minimum of 2 months	For non-cirrhotic patients 5-year post-treatment costs were 13-fold higher for non SVR patients vs. SVR (GBP 2,530 versus GBP 190), and 56-fold higher for non-SVR patients who were retreated (GBP 10,722)
Camma *et al*. 2012 (Italy) [87]	Treatment-naïve HCV genotype 1, aged 50 years with F2 fibrosis	Boceprevir- or telaprevir based triple therapy (including RGT) versus pegIFN plus ribavirin alone, time horizon of 20 years	ICER per SVR versus pegIFN plus ribavirin was EUR 56,960–85,650 for boceprevir and EUR 74,600–118,000 for telaprevir
Yfantopoulos *et al*. 2012 (Greece) [88]	Treatment-naïve and treatment-experienced HCV genotype 1	Telaprevir-based triple therapy versus boceprevir-based triple therapy	In total population, mean cost per SVR was EUR 46,635 for telaprevir and EUR 56,146 for boceprevir. For treatment-naïve population cost per SVR was EUR 38,868 and EUR 42,983, respectively. For treatment-experienced patients cost per SVR was EUR 48,966 and EUR 59,902 respectively. Telaprevir was dominant to boceprevir
Manos *et al*. 2013 (United States) [89]	Chronic HCV patients treated from 2002–2007, excluding pre- and post-liver transplant antiviral treatment	PegIFN plus ribavirin	In the 5 years following treatment mean yearly total (hospital and outpatient) costs in genotype 1 patients were USD 2,504 higher for non-responders than for patients with SVR (p = 0.042)

HCC, hepatocellular carcinoma; ICER, incremental cost-effectiveness ratio; RGT, response-guided therapy; SVR, sustained virologic response.

One 2013 US-based study Manos *et al*. examined follow up costs for patients achieving SVR versus non-responders over a 5-year period [89]. They report that patients with SVR (all genotypes) have mean annual costs (2007 USD) of USD 6,301 versus USD 10,149 for non-SVR patients, with the difference attributed to higher hospital costs (USD 5,167 versus USD 2,641) and outpatient costs (USD 4,983 versus USD 3,661). A similar UK-based analysis reported that costs in the 5 years post-treatment were 13-fold higher for patients who failed treatment versus those who achieved SVR, which increased to 56-fold for patients who initially failed treatment and were then retreated [85].

Three cost-effectiveness analyses presented results in terms of cost or incremental cost per SVR achieved [86-88]. In an Italian-based analysis Camma *et al*. reported an incremental cost per SVR achieved (versus pegIFN plus ribavirin) of EUR 60,500 per SVR for boceprevir IL28B guided therapy and EUR 74,600 per SVR for telaprevir IL28B guided therapy (2011 EUR) for treatment-naïve patients with HCV genotype 1. However, a key limitation of this analysis is that US pharmacy costs were used as Italian costs were not available at the time of the analysis, which may have led to under- or over-estimation of the true cost-effectiveness [87]. Another analysis from the Greek setting showed that for the overall HCV genotype 1 population (including treatment naïve patients and prior non-responders and relapsers), that telaprevir-based triple therapy was dominant to pegIFN plus ribavirin in terms of cost per SVR gained (telaprevir was associated with a cost-saving of EUR 10,403 per SVR gained) [88].

### Quality of life

The literature review process identified a total of 15 studies that examined HRQoL in patients with SVR [17,90-103], and a further study was identified via searches of the bibliographic sections of included studies [18]. The most commonly used instrument in HRQoL studies was the SF-36, and studies that used this almost universally showed that patients with SVR had better scores than non-responder/relapser/untreated populations, both in terms of sub-domains and physical and mental component summary scores, with a large proportion of between group differences achieving statistical significance. On an individual domain level, in studies that used the SF-36, the largest differences between patients with SVR and those without were reported for general health followed by role physical [18,103].

A total of seven studies (including two cost-effectiveness analyses of triple therapy), reported utility values for SVR using a number of different methods including standard gamble, time trade off (TTO) and the Health Utilities Index Mark 3 (HUI3) (Table 6). In one cost-effectiveness analysis by Liu *et al*. the mean utility value associated with SVR was dependent upon whether the subject had mild fibrosis or cirrhosis [104]. Previous studies have shown that HRQoL is influenced by disease severity, but the study by Liu *et al*. is one of the few studies to suggest the quality of life benefit of SVR is influenced by baseline disease severity. Utility values associated with the SVR state were strongly influenced by the method of assessment used and were typically highest using the TTO (ranging from 0.88–0.89) and standard gamble methods (0.86) (Table 6) and lowest using the SF-6D (0.71) and visual analog scale methods (0.74). Additionally, assessment of utility values using the EQ-5D valuation index led to values of 0.83–0.84 for SVR in comparison with 0.70–0.76 for non-response/relapse (Table 6) [95,99].

**Table 6:** **Health state utilities for HCV patients achieving SVR**

**Method**	**Study**	**Value**		**Difference**
		**SVR**	**Non-response/relapse**	
EQ5D	Thein *et al*. 2005 [93]	0.83	―	―
	Chong *et al*. 2003 [95]	0.83	0.76^c^	0.07
	Van Rooijen *et al*. 2011 [99]	0.84	0.70	0.14
SF-36	Thein *et al*. 2005 [93]	0.74–0.90	0.70–0.86^b^	0.04–0.05
SF-6D	Hsu *et al*. 2012 [103]	0.71	0.66^c^	0.05
	John-Baptiste *et al*. 2009 [18]	0.71	0.65	0.06
HUI3	Thein *et al*. 2005 [93]	0.77	―	―
	John-Baptiste *et al*. 2009 [18]	0.70	0.58	0.12
	Hsu *et al*. 2012 [103]	0.70	0.57^c^	0.13
HUI	Chong *et al*. 2003 [95]	0.77	0.73^c^	0.04
TTO	John-Baptiste *et al*. 2009 [18]	0.89	0.84	0.05
	Hsu *et al*. 2012 [103]	0.88	0.80^c^	0.08
SG	Thein *et al*. 2005 [93]	0.86	―	―
	Chong *et al*. 2003 [95]	0.86	0.79^c^	0.08
VAS	Thein *et al*. 2005 [93]	0.74	―	―
	Chong *et al*. 2003 [95]	0.74	0.70^c^	0.04
Not stated	Liu *et al*. 2012 [104]	0.933–1.00^a^	―	―
	Chhatwal *et al*. 2013 [105]	1.00	―	―

HUI3, Health Utilities Index Mark 3; SF-36, Medical Outcomes Study 36-item Short-Form Health Survey; SG, standard gamble; TTO, time trade off; VAS, visual analog scale.

^a^Age-specific quality of life weight.

^b^Untreated.

^c^Patients with mild/moderate HCV or chronic infection.

Most quality of life studies included in the review assessed HRQoL within the first year following treatment; however two studies assessed the impact of SVR at >3 years after completion of antiviral therapy. Both Mauss *et al*. [17] and John-Baptiste *et al*. [18] reported that the HRQoL benefits of SVR persist over >3 years, with both studies showing that patients with SVR had significantly better scores in all eight domains of the SF-36 in comparison with those who had failed treatment. Both Mauss *et al*. [17] and John-Baptiste *et al*. [18] also showed that SVR was associated with long-term benefits in terms of work productivity. Mauss *et al*. reported that a significantly higher proportion of patients who achieved SVR were employed (56%) in comparison with non-SVR patients (41%; p < 0.0001) [17]. Similarly, John-Baptiste *et al*. reported employment figures of 67% for patients with SVR versus 51% for those who failed treatment (p = 0.02). This analysis also showed that long-term work and leisure capacity were significantly compromised in treatment failures in comparison with the SVR group. Treatment failures had a mean (SD) reduction in work capacity of 5.8 (18)%, versus 1.1 (6)% for SVR; the corresponding figures for reduction in leisure capacity were 10.7 (24)% and 3.3 (13%), respectively [18].

## Discussion

The overarching aim of the present review was to consolidate published findings relating to the clinical, economic and quality of life benefits associated with achieving SVR and draw together these data to assess how clinical and quality of life benefits translate into economic benefits on both a per-patient and system-wide level. Previous research has largely focused on individual clinical, economic or quality of life aspects of SVR and has not examined how these benefits overlap and interact within a larger framework. For example, on an individual patient level, attainment of SVR is associated with lower risk of progression, HCC and liver-related mortality, less time spent in hospitals and improved symptoms and quality of life. However, when scaled up to a system wide level, SVR translates into substantial direct cost-savings for the payer due to costly complications avoided, as well as lower indirect costs due to lost productivity through absenteeism and presenteeism.

SVR is widely regarded as a cure and has been shown to be durable with rates of late relapse being in the region of 1–2%. In addition to halting progression of liver damage, SVR-induced regression of fibrosis and even cirrhosis has been reported. For example in a meta-analysis of 8 European studies, Veldt *et al*. reported regression of fibrosis in approximately one third of patients achieving SVR [106]. Additionally, risk factors such as heavy alcohol use or co-infection with hepatitis B may lead to progression of liver disease even in the presence of SVR. The clinical implications of potential low level viral persistence are not well characterized and it remains largely unknown whether it influences post-SVR progression of liver disease.

There is extensive evidence relating to the clinical benefits of SVR. A reduced risk for progression to cirrhosis, HCC, liver transplantation and liver-related mortality is evident regardless of setting, age, HCV subtype or level of fibrosis (Tables 2, 3, 4 and 7). However, the magnitude of the impact of SVR in terms of its impact on mortality rates varied notably between studies identified in this review, with some studies suggesting that following SVR the risk for liver-related mortality is comparable to that of the general population, whilst others suggest that mortality risk, although lower than for treatment failures, remains elevated in comparison with the general population. A contributing factor in this disparity may be heterogeneity in populations studied. Some studies excluded patients with advanced fibrosis or cirrhosis, whilst others were conducted exclusively in cirrhotic patients; there were also differences between patient populations in terms of age, previous treatment history, and the relative prevalence of different HCV genotypes.

**Table 7:** **Meta-analyses of long-term clinical outcomes in patients with SVR**

**Study**	**Details**	**Outcomes assessed**	**Key findings**
Almasio *et al*. 2003 [107]	Systematic review and pooled analysis (N = 1,031 patients for cirrhosis analysis, N = 3,914 patients for HCC analysis)	HCC, progression to cirrhosis	Risk reduction for progression to cirrhosis for SVR versus no SVR = −0.22 (−0.36 to −0.08). Risk reduction for HCC = −0.097 (−0.13 to −0.07)
Kimer *et al*. 2012 [51]	Systematic review and meta-analysis of 8 RCTs and 5 prospective studies (N = 3,208 patients); random effects model used. Patients treated with IFN, pegIFN or PegIFN plus ribavirin	HCC	RR (95% for HCC for SVR versus no intervention = 0.15 (0.05–0.45)
Morgan *et al.* 2013 [53]	Systematic review and meta-analysis of 30 studies (N = 31,528 patients)	HCC	For patients at all stages of disease HR (95% CI) for HCC for SVR versus non-response = 0.24 (0.18–0.31) (p < 0.001)
	(18 studies included in meta-analysis) investigating impact of treatment on risk for HCC		
			For patients with advanced liver disease HR (95% CI) for HCC for SVR versus non-response = 0.23 (0.16–0.35) (p < 0.001)
Singal *et al*. 2010 [49]	Systematic review and meta-analysis of 20 studies (N = 4,700 patients) in treatment-naïve patients treated with IFN or IFN plus ribavirin; random effects model used	HCC	RR (95% CI) for HCC for SVR versus non-responders = 0.35 (0.26–0.46)
Singal *et al*. 2010 [50]	Systematic review and meta-analysis of 26 studies (N = 13,191 patients)	HCC, hepatic decompensation, liver-related mortality	RR (95% CI) for SVR versus treatment failure were: HCC 0.21 (0.16–0.27) (p = ns) for all patients and 0.27 (0.19–0.39) (p = ns) for patients with cirrhosis.
			Liver-related mortality was 0.23 (0.10–0.52) (p = ns) for all patients and 0.13 (0.06–0.29) (p = ns) for patients with cirrhosis. RR (95% CI) Hepatic decompensation 0.16 (0.04–0.59) (p = ns) for all patients and 0.08 (0.03–0.21) (p = 0.02) for patients with cirrhosis

CI, confidence interval; HCC, hepatocellular carcinoma; RCT, randomized controlled trial; RR, relative risk.

The absolute risk and the magnitude of benefit does appear to be highly dependent on age and pre-treatment level of fibrosis. One study by Yoshida *et al*. [36] in the Japanese setting assessed the gain in HCC-free survival (defined as the difference in expected HCC-free survival with SVR versus without) according to age and fibrosis level. They report that the gain in HCC-free survival was greater when the subject was younger and had advanced fibrosis at baseline. For example for patients with stage F2 fibrosis the RR (95% CI) for HCC were 1.76 (0.47–6.67) for SVR versus 2.86 (1.59–5.13) for non-SVR, whereas for patients with F4 fibrosis the RRs (95% CI) increase to 4.78 (1.13–20.18) and 12.23 (6.81–21.95), respectively. A large proportion of the HCC studies identified in the current review (n = 11/24) were conducted in the Japanese setting, which has among the highest incidence of HCV-related HCC in the world, with an estimated 30,000 deaths per year attributable to HCC [36] and a mean annual treatment cost of USD 42,360 in Japan (2010 USD) [16]. As such, even a modest reduction in HCC, such as 100 cases avoided per year, would lead to savings of over USD 4 million for the payer.

The underlying reason for the high HCC rate in Japan is thought to be partly due to the high relative prevalence of genotype 1b (which is associated with a higher risk for HCC development in comparison with other genotypes [108]) relative to the US and Europe, and also to the fact that the spread of HCV is thought to have begun earlier in Japan than in Europe and North America, [109] therefore leading to an older prevalent population, with more advanced disease and therefore higher risk for developing HCC.

The clinical benefits of SVR are not limited to HCC. Patients with SVR have reduced risk of progression, liver-related mortality, liver transplantation and overall mortality in comparison with those not achieving SVR. Liver transplantation has a mean (global) cost of USD 146,960 in the year of transplant [16], so again even small reductions in the number of liver transplants required translate into substantial savings for the payer. The risk of overall mortality is reduced by approximately 5-fold, and liver related mortality approximately 10-fold, versus non-SVR, although this is influenced by age and level of fibrosis prior to treatment.

Patients with HCV have been shown to be at elevated risk for co-morbid conditions including type 2 diabetes [110]. Three studies showed that patients with SVR had a lower incidence of new onset diabetes versus non-responders. The mechanism for this is not clear, although hypotheses include elevated insulin resistance caused by pro-inflammatory cytokines [71]. It is has also proposed that insulin resistance may influence the likelihood of achieving SVR, rather than SVR influencing diabetogenic processes [111]. HCV is associated with a number of other extra-hepatic complications, although there are a lack of data on the impact of SVR on these.

The clinical benefits associated with SVR due to complications avoided translate into economic benefits from a third party perspective. The magnitude of economic benefit is difficult to quantify, due to uncertainty of prevalence estimates and continued advances in therapy leading to ongoing improvements in SVR rates but owing to the high cost, even a small reduction in the incidence of HCC would have considerable economic implications. In addition to direct costs, the attainment of SVR also has implications on indirect costs such as lost productivity, with evidence to suggest that employment rates are higher amongst patients with SVR versus those without [17,18].

The clinical benefits associated with the achievement of SVR translate into clinically meaningful benefits for patients by improving symptoms, functioning and health related quality of life, compared with those not able to achieve SVR. The findings of quality of life studies consistently showed that patients with SVR had higher utility values and SF-36 and EQ-5D scores in comparison with those who did not respond to treatment. However, in the literature review it was noted that there is a paucity of quality of life studies with long-term follow-up (≥5 years). Although SVR leads to improved quality of life in the short-term, data relating to whether or not this improvement persists in the long term are lacking.

Although the scope of the present review was such that the endpoints of fatigue and depression were not assessed directly, SVR is also associated with other benefits in terms of patient reported outcomes including fatigue and depression, which are common side effects associated with antiviral treatment. The Fatigue Severity Scale (FSS) is a commonly used instrument to assess fatigue in HCV studies. The FSS has good reliability, validity and responsiveness and a total score ≥4 is indicative of severe fatigue. In addition to improved SVR, protease inhibitors are associated with benefits in terms of reduced fatigue. Published data relating to the magnitude of change in FSS score required to constitute a minimally important difference are lacking. However, analysis of phase III simeprevir trial data indicate that a clinically meaningful change (improvement or worsening) may be as small as 0.33–0.34 and that patients with SVR have significant improvements in FSS score versus non-responders (Janssen, data on file).

The current study has several limitations that should be acknowledged with regard to interpretation of the findings. In particular, the review included studies that compare SVR groups with both untreated groups and non-responder groups. Several studies in Japan have shown that risk of HCC and overall mortality are reduced, although not significantly in patients who receive treatment but fail to achieve SVR in comparison with untreated patients, although the mechanism behind this is poorly understood [57,112]. Moreover, in a considerable proportion of the studies reported here no distinction is made by the authors in the non-SVR groups in terms of null-response, partial response or relapse following treatment. The potential benefits of SVR in relapsers is an area that warrants further investigation as two studies included here suggested that patients who relapse have lower risks for overall mortality and HCC in comparison with true non-responders [37]. Similarly, whether benefits of SVR are different across different sub-populations, such as patients with hepatitis B or HIV coinfection, or is influenced by genotype, is an issue for future analysis. A further limitation of the current review is that no formal quality assessment of included studies was performed.

While this systematic literature review attempted to be as holistic as possible in capturing the impact of achieving SVR in patients chronically infected with hepatitis C, it was not possible to capture all possible consequences. For example, the benefits associated with reduced infection risk were not considered, and therefore represent a limitation of the review. Additionally, during the literature search it was noted that an aspect of HCV that is often overlooked in the literature is the stigma associated with HCV and the impact of this on patients’ quality of life, disclosure practices and treatment-seeking behavior. Stigma may be subtle and is inherently difficult to quantify. One of the key factors in stigma arises due to fear of transmission, which although limited to blood-borne routes, does not prevent stigma. Patients with SVR are no longer at risk of transmitting HCV to others, therefore the stigma associated with HCV should be removed. Another aspect to consider is the public health benefit associated with a lower population prevalence; a reduced population prevalence means that there are fewer people from whom HCV can be transmitted to others.

## Conclusions

In conclusion, review of the literature has shown that achievement of SVR in patients with chronic HCV infection is associated with significant clinical, economic and quality of life benefits. Patients who achieve SVR, including those with advanced disease, have a substantially reduced risk of progression to cirrhosis, development of HCC and both liver-related and all cause mortality. This reduced risk of late stage complications also leads to economic benefits. Post-treatment, patients with SVR also have lower healthcare resource utilization versus non-responders, which also translates into substantial economic benefits from a healthcare payer perspective. Finally, the attainment of SVR is also associated with improved quality of life.

## Additional file

Additional file 1:
**PRISMA Flow Diagram: Achieving sustained virologic response in hepatitis C: a review of the clinical, economic and quality of life benefits.**
