# Supplementary material for: Achieving sustained virologic response in hepatitis C: a systematic review of the clinical, economic and quality of life benefits
Source: BMC Infect Dis. 2015 Jan 17;15:19. doi: 10.1186/s12879-015-0748-8 (PMC4299677; doi:10.1186/s12879-015-0748-8)
Supplement: Additional file 1: — PRISMA Flow Diagram: Achieving sustained virologic response in hepatitis C: a review of the clinical, economic and quality of life benefits. [file 12879_2015_748_MOESM1_ESM.doc]

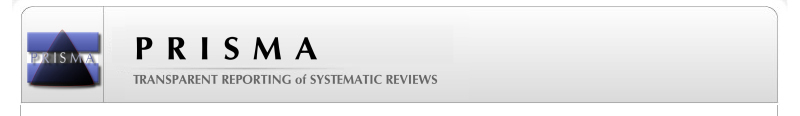
**PRISMA 2009 Flow Diagram: Achieving sustained virologic response in hepatitis C: a review of the clinical, economic and quality of life benefits**

**Screening**

**Included**

**Eligibility**

**Identification**

Records identified through database searching^a^
(n=6,564)

Additional records identified through other sources
(n=1)

Records after duplicates removed
(n=4,207)

Records screened
(n=4,207)

Records excluded
(n=3,911)

Full-text articles assessed for eligibility
(n=296)

Studies included in qualitative synthesis
(n=62)

Studies included in quantitative synthesis (meta-analysis)
(n=0)^c^

Full-text articles excluded, with reasons
(n=234)

- Not reporting outcomes of interest/not dealing with SVR (n=183)
- Wrong publication type (n=16)^b^
- Not chronic HCV/not HCV monoinfection (n=8)
- <1 year follow up (n=2)
- <100 patients (n=25)

^a^Searches were performed using the EMBASE, PubMed and Cochrane Library databases

^b^Publications that were classed as case studies, letters, editorials, notes or commentaries were excluded

^c^No meta-analysis was performed

**Note:** the original literature searches were re-run in April 2014 to capture publications published since the original searches. A total of twenty additional clinical articles and three additional economic studies were identified.
